# Supplementary material for: Subset scanning for multi-trait analysis using GWAS summary statistics
Source: Bioinformatics. 2024 Jan 5;40(1):btad777. doi: 10.1093/bioinformatics/btad777 (PMC11087659; doi:10.1093/bioinformatics/btad777)
Supplement: btad777_Supplementary_Data [file btad777_supplementary_data.pdf]

Supplementary Materials for Subset scanning for multi-trait analysis using GWAS summary statistics

Rui Cao[1], Evan Olawsky[1], Edward McFowland III[2], Erin Marcotte[3], Logan Spector[3], and Tianzhong Yang[1,3]

[1]Division of Biostatistics and Health Data Science, University of Minnesota
[2]Technology and Operations Management, Harvard Business School
[3]Department of Pediatrics, University of Minnesota

1 Method

1.1 Strong Linear Time Subset Scanning Property

Our subset scanning algorithm relies on a score statistic F, which is a function of a non-empty subset $S \subseteq \{1, \ldots, p\}$. It quantifies the amount of anomalousness found in traits $\{y_j | j \in S\}$ under the null hypothesis that no trait is associated with the SNP. The most anomalous subset is found by maximizing $F(S)$ over all non-empty subsets of the traits. Calculating $F(S)$ over all possible subsets S is extremely burdensome when p is large. To ensure efficient maximization, we use subset scanning techniques and strive for a statistic accompanying priority function that satisfies the strong linear time subset scanning (LTSS) property:

Definition 1 (Neill (2012))**.** *The score function $F(S)$ and priority function $G(j; \tilde{y})$ satisfy the strong LTSS property if and only if, for all $j = 1, \ldots, p$, $\max_{S:|S|=j} F(S) = F(\{\tilde{y}^{(1)}, \ldots, \tilde{y}^{(j)}\})$, where $\tilde{y}^{(j)}$ is the trait with the jth highest value of $G(\cdot; \tilde{y})$.*

If $F(S)$ satisfies the strong LTSS property, the subset S^* that maximizes $F(S)$ must be the subset containing the c highest-priority traits $\{\tilde{y}^{(1)}, \ldots, \tilde{y}^{(c)}\}$ for some c between 1 and p. Thus, to solve the global optimization problem, we can simply sort the traits by their priority value given by G and then compute $F(S)$ with S taken to be one of the p subsets $\{\tilde{y}^{(1)}\}, \{\tilde{y}^{(1)}, \tilde{y}^{(2)}\}, \ldots, \{\tilde{y}^{(1)}, \ldots, \tilde{y}^{(p)}\}$.

Neill (2012) gave a constructive theorem that produces a specific priority function $G(j; \tilde{Y})$ that follows directly from the score function $F(S)$ when certain properties hold. This pair of functions is then guaranteed to satisfy the strong LTSS property.

Theorem 1 (Neill (2012))**.** *Let $F(S) = F(T, |S|)$ be a function of one additive statistic of subset S, $T(S) = \sum_{j \in S} g(j; \tilde{y})$ (where $g(j; \tilde{y})$ depends only on trait $\tilde{y}_j$) and the cardinality*

of S, $|S|$. Assume that $F(S)$ is monotonically increasing with $T(S)$, then $F(S)$ satisfies the strong LTSS property with priority function $G(j;\tilde{y}) = g(j;\tilde{y})$.

As demonstrated in the main text, for the HC statistic, it can be easily seen that $F_{HC}(S)$ is a function of $||S||$ and one additive statistic N_α. $F_{HC}(S)$ is monotonically increasing with N_α and thus satisfies the strong LTSS property with priority function $G_{HC,\alpha}(p_j^*) = I(p_j^* < \alpha)$. Similarly, the TC statistic also satisfies the strong LTSS property. Both the HC and TC have a linear scan time over the number of traits.

1.2 Significance of HC

In this section, we will show the derivation of the analytical HC p-values of the HC statistic. As $F_{HC,\alpha}(S)$ is an decreasing function of $|S|$, for $\alpha = \{\alpha_1, \alpha_2, ..., \alpha_m\}$,

$$\begin{aligned} p_{HC} &= Pr(\max_\alpha \max_S F_{HC,\alpha}(S) \geqslant h_{HC}|H_0) \\ &= Pr(\max_\alpha \sqrt{\frac{1-\alpha}{\alpha}} N_\alpha \geqslant h_{HC}|H_0) \\ &= 1 - Pr(\max_\alpha \sqrt{\frac{1-\alpha}{\alpha}} N_\alpha < h_{HC}|H_0) \\ &= 1 - Pr(\bigcap_i^m \sqrt{\frac{1-\alpha_i}{\alpha_i}} N_{\alpha_i} < h_{HC}|H_0) \end{aligned} \tag{1}$$

Lemma 1. *For $0 < \alpha_{(1)} < \alpha_{(2)} < ... < \alpha_{(m)} < 1$ and $j = 1, 2, ..., p$, $p_j^* \overset{iid}{\sim} Uniform(0, 1)$ under the H_0. $N_{\alpha_{(i)}} = \sum_{j=1}^p I(p_j^* < \alpha_{(i)})$ follows a Binomial(p, $\alpha_{(i)}$) distribution. Moreover, $N_{\alpha_{(i)}}|N_{\alpha_{(i+1)}} \sim Binomial(N_{\alpha_{(i+1)}}, \frac{\alpha_{(i)}}{\alpha_{(i+1)}})$, which is independent of $N_{\alpha_{(i+2)}}$, $N_{\alpha_{(i+3)}}$,...,$N_{\alpha_{(m)}}$.*

Under the null,

$$\begin{aligned} Pr(\bigcap_i^m \sqrt{\frac{1-\alpha_i}{\alpha_i}} N_{\alpha_i} < h_{HC}) = & Pr(\sqrt{\frac{1-\alpha_{(m)}}{\alpha_{(m)}}} N_{\alpha_{(m)}} < h_{HC}) \\ & Pr(\sqrt{\frac{1-\alpha_{(m-1)}}{\alpha_{(m-1)}}} N_{\alpha_{(m-1)}} < h_{HC}|\sqrt{\frac{1-\alpha_{(m)}}{\alpha_{(m)}}} N_{\alpha_{(m)}} < h_{HC}) \\ & Pr(\sqrt{\frac{1-\alpha_{(m-2)}}{\alpha_{(m-2)}}} N_{\alpha_{(m-2)}} < h_{HC}|\bigcap_i^m \sqrt{\frac{1-\alpha_{(i)}}{\alpha_{(i)}}} N_{\alpha_{(i)}} < h_{HC}) \\ & ... \\ & Pr(\sqrt{\frac{1-\alpha_{(1)}}{\alpha_{(1)}}} N_{\alpha_{(1)}} < h_{HC}|\bigcap_i^m \sqrt{\frac{1-\alpha_{(i)}}{\alpha_{(i)}}} N_{\alpha_{(i)}} < h_{HC}) \end{aligned}$$

We proposed the following algorithm to estimate p_{HC}:

Step 1: Calculate the marginal probabilities $Pr(N_{\alpha_{(m)}} = 1)$, ... ,$Pr(N_{\alpha_{(m)}} = \lceil \frac{\alpha_{(m)}}{1-\alpha_{(m)}} H_{HC}^2 - 1 \rceil)$

Step 2: When $i = m-1$, calculate probabilities

$$\begin{aligned} & Pr(N_{\alpha_{(i)}} = j | N_{\alpha_{(i+1)}} < \frac{\alpha_{(i+1)}}{1-\alpha_{(i+1)}} h_{HC}^2) \\ = & \frac{Pr(\{N_{\alpha_{(i)}} = j\} \bigcap \{N_{\alpha_{(i+1)}} < \frac{\alpha_{(i+1)}}{1-\alpha_{(i+1)}} h_{HC}^2\})}{Pr(N_{\alpha_{(i+1)}} < \frac{\alpha_{(i+1)}}{1-\alpha_{(i+1)}} h_{HC}^2)} \\ = & \frac{\sum_{k=j}^{\lceil \frac{\alpha_{(i+1)}}{1-\alpha_{(i+1)}} h_{HC}^2 - 1 \rceil} Pr(\{N_{\alpha_{(i)}} = j\} \bigcap \{N_{\alpha_{(i+1)}} = k\})}{\sum_{k=j}^{\lceil \frac{\alpha_{(i+1)}}{1-\alpha_{(i+1)}} h_{HC}^2 - 1 \rceil} Pr(N_{\alpha_{(i+1)}} = k)} \end{aligned}$$

for each $0 \le j < \frac{\alpha_{(i)}}{1-\alpha_{(i)}} h_{HC}^2$.

Step 3: When $i = m-2, ..., 1$, substitute probabilities of $N_{\alpha_{(i+1)}}$ by conditional probabilities, and $Pr(N_{\alpha_{(i)}} = j | \bigcup_{l=i+1}^{m} \{N_{\alpha_{(l)}} < \frac{\alpha_{(l)}}{1-\alpha_{(l)}} h_{HC}^2\})$ can be derived in sequential order by using the following method:

$$\begin{aligned} & Pr(N_{\alpha_{(i)}} = j | \bigcup_{l=i+1}^{m} \{N_{\alpha_{(l)}} < \frac{\alpha_{(l)}}{1-\alpha_{(l)}} h_{HC}^2\}) \\ = & \frac{Pr(\{N_{\alpha_{(i)}} = j\} \bigcup \{N_{\alpha_{(i+1)}} < \frac{\alpha_{(i+1)}}{1-\alpha_{(i+1)}} h_{HC}^2\} | \bigcup_{l=i+2}^{m} \{N_{\alpha_{(l)}} < \frac{\alpha_{(l)}}{1-\alpha_{(l)}} h_{HC}^2\})}{Pr(N_{\alpha_{(i+1)}} < \frac{\alpha_{(i+1)}}{1-\alpha_{(i+1)}} h_{HC}^2) | \bigcup_{l=i+2}^{m} \{N_{\alpha_{(l)}} < \frac{\alpha_{(l)}}{1-\alpha_{(l)}} h_{HC}^2\}} \\ = & \frac{\sum_{k=j}^{\lceil \frac{\alpha_{(i+1)}}{1-\alpha_{(i+1)}} h_{HC}^2 - 1 \rceil} Pr(\{N_{\alpha_{(i)}} = j\} \bigcup \{N_{\alpha_{(i+1)}} = k\} | \bigcup_{l=i+2}^{m} \{N_{\alpha_{(l)}} < \frac{\alpha_{(l)}}{1-\alpha_{(l)}} h_{HC}^2\})}{\sum_{k=j}^{\lceil \frac{\alpha_{(i+1)}}{1-\alpha_{(i+1)}} h_{HC}^2 - 1 \rceil} Pr(N_{\alpha_{(i+1)}} = k | \bigcup_{l=i+2}^{m} \{N_{\alpha_{(l)}} < \frac{\alpha_{(l)}}{1-\alpha_{(l)}} h_{HC}^2\})} \\ = & \frac{\begin{array}{r} \sum_{k=j}^{\lceil \frac{\alpha_{(i+1)}}{1-\alpha_{(i+1)}} h_{HC}^2 - 1 \rceil} Pr(N_{\alpha_{(i)}} = j | N_{\alpha_{(i+1)}} = k, \bigcup_{l=i+2}^{m} \{N_{\alpha_{(l)}} < \frac{\alpha_{(l)}}{1-\alpha_{(l)}} h_{HC}^2\}) \\ Pr(N_{\alpha_{(i+1)}} = k | \bigcup_{l=i+2}^{m} \{N_{\alpha_{(l)}} < \frac{\alpha_{(l)}}{1-\alpha_{(l)}} h_{HC}^2\}) \end{array}}{\sum_{k=j}^{\lceil \frac{\alpha_{(i+1)}}{1-\alpha_{(i+1)}} h_{HC}^2 - 1 \rceil} Pr(N_{\alpha_{(i+1)}} = k | \bigcup_{l=i+2}^{m} \{N_{\alpha_{(l)}} < \frac{\alpha_{(l)}}{1-\alpha_{(l)}} h_{HC}^2\})} \end{aligned}$$

by Lemma 1,

$$= \frac{\sum_{k=j}^{\lceil \frac{\alpha_{(i+1)}}{1-\alpha_{(i+1)}} h_{HC}^2 - 1 \rceil} Pr(N_{\alpha_{(i)}} = j | N_{\alpha_{(i+1)}} = k) Pr(N_{\alpha_{(i+1)}} = k | \bigcup_{l=i+2}^{m} \{N_{\alpha_{(l)}} < \frac{\alpha_{(l)}}{1-\alpha_{(l)}} h_{HC}^2\})}{\sum_{k=j}^{\lceil \frac{\alpha_{(i+1)}}{1-\alpha_{(i+1)}} h_{HC}^2 - 1 \rceil} Pr(N_{\alpha_{(i+1)}} = k | \bigcup_{l=i+2}^{m} \{N_{\alpha_{(l)}} < \frac{\alpha_{(l)}}{1-\alpha_{(l)}} h_{HC}^2\})}$$

for each $0 \le j < \frac{\alpha_{(i)}}{1-\alpha_{(i)}} h_{HC}^2$.

Step 4: For $i = m-1, ..., 1$

$$
\begin{aligned}
& Pr(N_{\alpha_{(i)}} < \frac{\alpha_{(i)}}{1-\alpha_{(i)}} h^2_{HC} | N_{\alpha_{(i+1)}} < \frac{\alpha_{(i+1)}}{1-\alpha_{(i+1)}} h^2_{HC}) \\
= & \sum_{j=0}^{\lceil \frac{\alpha_{(i)}}{1-\alpha_{(i)}} h^2_{HC} - 1 \rceil} Pr(N_{\alpha_{(i)}} = j | N_{\alpha_{(i+1)}} < \frac{\alpha_{(i+1)}}{1-\alpha_{(i+1)}} h^2_{HC})
\end{aligned}
$$

1.3 Choice of α grid in HC and TC

Both HC and TC statistics follow a linear scan time given a fixed number of $\alpha's$. In implementation, we recommend a grid of 200 $\alpha's$ with the minimum α as the Bonferroni significant p-value cutoff and maximum α of 0.05. A practical issue faced by TraitScan and other threshold-based multi-trait methods is the choice of the density of thresholds. For the statistic S_{het}, Zhu et al. (2015) recommended flexible thresholds which are the same as the input z-scores, while Bu et al. (2020) and TraitScan used fixed p-value thresholds. In simulations (not shown), we observed a dramatic power and precision loss of S_{het} using the same fixed threshold grid as in TraitScan. It is noteworthy that TraitScan always selects traits that are statistically significant in the decorrelated univariate analysis as we set the minimum α as the Bonferroni significant p-value cutoff. Based on our experience with simulations and real data analyses, we recommend a maximum α of 0.05 as traits with decorrelated p-values larger than 0.05 have never been selected by our algorithm. Since TraitScan includes a MC step simulating the null distribution of test statistics, an overly dense α grid may slow down the algorithm. We found a grid of 200 $\alpha's$ is sufficiently dense when handling hundreds of GWAS traits.

2 Real data analysis results

Table S1: EWS SNPs and UK Biobank traits

SNP	Traits - Bonferroni	Traits - TraitScan (minp)	UK Biobank p-values	TraitScan decorrelated p-values
rs113663169	Natural Hair color: blonde	Natural Hair color: blonde	$\leq 10^{-20}$	$\leq 10^{-20}$
	Natural Hair color: dark brown	Natural Hair color: dark brown	2.22×10^{-16}	2.08×10^{-12}
	Natural Hair color: black	Natural Hair color: black	2.34×10^{-8}	2.94×10^{-7}
	Facial ageing	Facial ageing	1.09×10^{-5}	7.36×10^{-6}
	Standing height		2.28×10^{-8}	
	Sitting height *		9.63×10^{-8}	
	Seated height		3.19×10^{-7}	
	Comparative height size at age 10		3.51×10^{-7}	
rs7742053		Alcohol usually taken with meals	7.67×10^{-3}	3.34×10^{-3}
		Fruit consumers (recent 24 hours)	2.57×10^{-2}	4.71×10^{-3}
		Treatment speciality of consultant (recoded): Geriatric medicine	9.77×10^{-3}	7.88×10^{-3}
		Inguinal/femoral hernia repair	1.25×10^{-2}	8.27×10^{-3}
		Glucose (blood)	1.32×10^{-2}	8.53×10^{-3}
		Headaches for 3+ months	2.05×10^{-2}	1.00×10^{-2}
		Inpatient record: K63.4 **	3.95×10^{-2}	1.31×10^{-2}
		Tea intake (cups per day)	2.71×10^{-2}	1.31×10^{-2}
		Lifetime number of sexual partners	4.51×10^{-2}	1.46×10^{-2}
		Hospital episode type: General episode	7.01×10^{-2}	1.47×10^{-2}
		Chest pain or discomfort	1.90×10^{-2}	1.48×10^{-2}
		Naan bread intake	2.47×10^{-2}	1.52×10^{-2}
		Risk taking	4.01×10^{-2}	1.52×10^{-2}
rs10822056	Standing height	Standing height	3.22×10^{-14}	2.73×10^{-13}
	Platelet crit (blood)	Platelet crit (blood)	1.07×10^{-13}	7.70×10^{-10}
	Cystatin C (blood)	Cystatin C (blood)	2.51×10^{-9}	8.53×10^{-9}
	Total protein (blood)	Total protein (blood)	3.60×10^{-7}	1.10×10^{-8}
	Monocyte count (blood)	Monocyte count (blood)	1.20×10^{-13}	3.96×10^{-8}
	Nap during day	Nap during day	2.49×10^{-7}	1.48×10^{-6}
		Alkaline phosphatase (blood)	5.62×10^{-5}	2.17×10^{-6}
	Monocyte percentage (blood)	Monocyte percentage (blood)	7.81×10^{-6}	6.53×10^{-6}
	Platelet distribution width (blood)	Platelet distribution width (blood)	3.04×10^{-8}	9.34×10^{-6}
	Bread intake	Bread intake	3.80×10^{-6}	1.05×10^{-5}
	Appendicectomy	Appendicectomy	3.50×10^{-6}	1.50×10^{-5}
	Platelet count (blood)		8.54×10^{-11}	
	Lymphocyte count (blood)		4.84×10^{-9}	
	White blood cell (leukocyte) count		6.73×10^{-8}	
	Ease of skin tanning		3.46×10^{-6}	
	Comparative height size at age 10		9.23×10^{-6}	
rs2412476	Red blood cell (erythrocyte) distribution width (blood)	Red blood cell (erythrocyte) distribution width (blood)	$\leq 10^{-20}$	2.00×10^{-15}
	Aspartate aminotransferase (blood)	Aspartate aminotransferase (blood)	$\leq 10^{-20}$	1.57×10^{-13}
	Albumin (blood)	Albumin (blood)	3.96×10^{-13}	1.10×10^{-10}
	Alanine aminotransferase (blood)	Alanine aminotransferase (blood)	1.33×10^{-15}	4.05×10^{-10}
	Red blood cell (erythrocyte) count (blood)	Red blood cell (erythrocyte) count (blood)	5.67×10^{-10}	4.93×10^{-7}
	Mean corpuscular haemoglobin (blood)		2.56×10^{-8}	
	Gamma glutamyltransferase (blood)		1.16×10^{-7}	
	Reticulocyte percentage (blood)		1.03×10^{-6}	
		High light scatter reticulocyte percentage (blood)	4.97×10^{-6}	
	Haematocrit percentage (blood)	Haematocrit percentage (blood)	9.01×10^{-6}	
rs6047482	Urea (blood)	Urea (blood)	1.02×10^{-7}	5.65×10^{-10}
		Insulin-like growth factor 1 (blood)	1.68×10^{-4}	3.60×10^{-6}
rs6106336	Insulin-like growth factor 1	Insulin-like growth factor 1 (blood)	6.16×10^{-7}	2.11×10^{-7}
		Red blood cell (erythrocyte) distribution width (blood)	8.25×10^{-4}	1.26×10^{-4}
		Knee pain experienced in last month	7.57×10^{-3}	8.81×10^{-4}
	Sitting height *	Sitting height *	5.54×10^{-6}	9.71×10^{-4}
		Age first had sexual intercourse	1.32×10^{-2}	1.21×10^{-3}
		Facial aging	9.44×10^{-3}	1.98×10^{-3}
		Average total household income before tax	1.52×10^{-2}	2.42×10^{-3}
	Seated height	Seated height	6.05×10^{-6}	3.15×10^{-3}

		Coffee intake	8.10×10^{-3}	3.52×10^{-3}

* Difference between seated height and seating box height

** Diagnostic endoscopic examination of lower bowel and biopsy of lesion of lower bowel using fibreoptic sigmoidoscope.

Table S2: Bidirectional MR analysis on traits selected by TraitScan

UK Biobank Traits	Trait as exposure or outcome	Number of instru-mental SNPs	MR method	beta	se(beta)	p-value
Hair color (natural, before greying): Black	Exposure	27	MR Egger	0.3989	4.6805	0.9328
			Weighted median	3.6525	3.5257	0.3002
			Inverse variance weighted	-1.9314	2.5144	0.4424
			Simple mode	-10.6567	6.5940	0.1181
			Weighted mode	4.7074	4.6294	0.3186
	Outcome	44	MR Egger	0.0018	0.0010	0.0846
			Weighted median	-0.0004	0.0004	0.3075
			Inverse variance weighted	0.0000	0.0003	0.9135
			Simple mode	-0.0014	0.0009	0.1253
			Weighted mode	-0.0012	0.0008	0.1423
Hair color (natural, before greying): Blonde	Exposure	76	MR Egger	0.7295	1.3795	0.5985
			Weighted median	1.3325	1.3554	0.3255
			Inverse variance weighted	0.3980	0.8205	0.6276
			Simple mode	2.8852	2.6004	0.2707
			Weighted mode	1.5777	1.4056	0.2653
	Outcome	44	MR Egger	-0.0034	0.0021	0.1056
			Weighted median	-0.0005	0.0005	0.3400
			Inverse variance weighted	-0.0009	0.0006	0.1535
			Simple mode	-0.0006	0.0011	0.5793
			Weighted mode	-0.0007	0.0011	0.5362
Hair color (natural, before greying): Dark brown	Exposure	68	MR Egger	-2.2877	1.6478	0.1697
			Weighted median	-0.7039	1.0521	0.5035
			Inverse variance weighted	0.2969	0.7277	0.6833
			Simple mode	-2.1311	1.9210	0.2712
			Weighted mode	-0.6626	1.3130	0.6155
	Outcome	44	MR Egger	0.0055	0.0028	0.0544
			Weighted median	0.0007	0.0009	0.4503
			Inverse variance weighted	0.0011	0.0008	0.1833
			Simple mode	-0.0034	0.0024	0.1619
			Weighted mode	0.0028	0.0026	0.2930
Facial aging	Exposure	50	MR Egger	-0.8922	2.3261	0.7030
			Weighted median	-1.7800	2.0270	0.3799
			Inverse variance weighted	-1.5744	1.1366	0.1660
			Simple mode	-6.3617	4.0535	0.1230
			Weighted mode	-1.6605	2.2010	0.4542
	Outcome	43	MR Egger	0.0056	0.0022	0.0173
			Weighted median	0.0018	0.0008	0.0155
			Inverse variance weighted	0.0015	0.0007	0.0330
			Simple mode	0.0023	0.0017	0.1980
			Weighted mode	0.0023	0.0016	0.1516
Cystatin C (blood)	Exposure	166	MR Egger	0.1292	0.7313	0.8600
			Weighted median	1.1117	0.4356	0.0107
			Inverse variance weighted	0.5494	0.2856	0.0544
			Simple mode	0.4599	1.0456	0.6606
			Weighted mode	0.9985	0.6822	0.1452
	Outcome	44	MR Egger	-0.0020	0.0051	0.7044
			Weighted median	0.0004	0.0015	0.8140
			Inverse variance weighted	-0.0003	0.0015	0.8271
			Simple mode	0.0013	0.0031	0.6858
			Weighted mode	0.0012	0.0029	0.6954
Total protein (blood)	Exposure	152	MR Egger	-0.1554	0.5907	0.7929
			Weighted median	-0.1080	0.4216	0.7979
			Inverse variance weighted	-0.6338	0.2507	0.0115
			Simple mode	-0.4577	1.1791	0.6984
			Weighted mode	0.6880	0.6947	0.3236
	Outcome	44	MR Egger	-0.0010	0.0068	0.8872
			Weighted median	0.0000	0.0018	0.9927
			Inverse variance weighted	0.0003	0.0020	0.8994
			Simple mode	-0.0005	0.0039	0.9014
			Weighted mode	-0.0009	0.0041	0.8314
Appendicectomy	Exposure	12	MR Egger	18.1444	13.7169	0.2154
			Weighted median	-7.9528	5.5831	0.1543
			Inverse variance weighted	-4.5269	4.0213	0.2603

			Simple mode	-9.0879	8.9991	0.3343
			Weighted mode	-8.9492	7.5055	0.2582
	Outcome	43	MR Egger	-0.0006	0.0013	0.6457
			Weighted median	0.0001	0.0005	0.8165
			Inverse variance weighted	0.0003	0.0004	0.4689
			Simple mode	0.0004	0.0010	0.6500
			Weighted mode	0.0002	0.0008	0.7953
Nap during day	Exposure	48	MR Egger	5.3074	5.3667	0.3279
			Weighted median	1.3267	1.7947	0.4598
			Inverse variance weighted	0.0157	1.2686	0.9901
			Simple mode	3.2890	4.4047	0.4590
			Weighted mode	4.1869	3.2736	0.2072
	Outcome	44	MR Egger	-0.0026	0.0027	0.3506
			Weighted median	0.0009	0.0009	0.3140
			Inverse variance weighted	0.0010	0.0008	0.2316
			Simple mode	0.0017	0.0020	0.3909
			Weighted mode	0.0013	0.0018	0.4641
Bread intake	Exposure	15	MR Egger	-18.6268	10.9839	0.1137
			Weighted median	1.4511	2.0440	0.4778
			Inverse variance weighted	0.7143	1.5729	0.6498
			Simple mode	3.0283	4.3717	0.4998
			Weighted mode	2.4067	3.8195	0.5388
	Outcome	43	MR Egger	-0.0054	0.0040	0.1906
			Weighted median	-0.0020	0.0013	0.1357
			Inverse variance weighted	-0.0026	0.0012	0.0323
			Simple mode	-0.0027	0.0027	0.3188
			Weighted mode	-0.0013	0.0023	0.5648
Platelet crit	Exposure	242	MR Egger	-0.1474	0.4018	0.7140
			Weighted median	0.2041	0.3049	0.5033
			Inverse variance weighted	0.1448	0.1914	0.4493
			Simple mode	-0.2604	0.7592	0.7319
			Weighted mode	0.4385	0.5544	0.4298
	Outcome	44	MR Egger	0.0037	0.0052	0.4852
			Weighted median	-0.0003	0.0016	0.8269
			Inverse variance weighted	0.0010	0.0015	0.4958
			Simple mode	0.0006	0.0033	0.8581
			Weighted mode	0.0015	0.0027	0.5762
Platelet distribution width	Exposure	183	MR Egger	0.2714	0.3210	0.3989
			Weighted median	0.3991	0.2970	0.1790
			Inverse variance weighted	-0.0267	0.1807	0.8823
			Simple mode	0.4277	0.7708	0.5797
			Weighted mode	0.9996	0.4172	0.0176
	Outcome	44	MR Egger	-0.0122	0.0070	0.0902
			Weighted median	0.0019	0.0017	0.2722
			Inverse variance weighted	0.0011	0.0022	0.6073
			Simple mode	0.0032	0.0036	0.3766
			Weighted mode	0.0030	0.0037	0.4231
Monocyte count	Exposure	186	MR Egger	0.2082	0.3686	0.5728
			Weighted median	0.1836	0.2863	0.5215
			Inverse variance weighted	0.1908	0.1895	0.3140
			Simple mode	0.5472	0.6132	0.3734
			Weighted mode	0.3017	0.3743	0.4212
	Outcome	44	MR Egger	0.0016	0.0062	0.7995
			Weighted median	-0.0029	0.0016	0.0764
			Inverse variance weighted	-0.0004	0.0018	0.8448
			Simple mode	-0.0082	0.0038	0.0379
			Weighted mode	-0.0063	0.0033	0.0588
Monocyte percentage	Exposure	170	MR Egger	-0.2788	0.4337	0.5212
			Weighted median	-0.0674	0.3257	0.8360
			Inverse variance weighted	0.1279	0.2093	0.5411
			Simple mode	-0.2530	0.6413	0.6937
			Weighted mode	-0.3093	0.4654	0.5072
	Outcome	44	MR Egger	0.0047	0.0044	0.2983
			Weighted median	-0.0021	0.0015	0.1847
			Inverse variance weighted	-0.0002	0.0013	0.8990
			Simple mode	-0.0039	0.0032	0.2224
			Weighted mode	-0.0039	0.0028	0.1644
Standing height	Exposure	440	MR Egger	0.1577	0.4587	0.7312
			Weighted median	-0.2046	0.3391	0.5463

			Inverse variance weighted	-0.0678	0.2088	0.7452
			Simple mode	-0.4919	0.9751	0.6142
			Weighted mode	-0.3131	0.7779	0.6875
	Outcome	43	MR Egger	-0.0023	0.0050	0.6582
			Weighted median	0.0003	0.0011	0.7656
			Inverse variance weighted	-0.0001	0.0015	0.9355
			Simple mode	-0.0017	0.0029	0.5645
			Weighted mode	-0.0019	0.0029	0.5100
Alkaline phosphatase	Exposure	191	MR Egger	-0.9791	0.3779	0.0104
			Weighted median	-1.1522	0.3217	0.0003
			Inverse variance weighted	-0.6501	0.2118	0.0021
			Simple mode	0.3132	0.7591	0.6804
			Weighted mode	-1.2185	0.3213	0.0002
	Outcome	44	MR Egger	-0.0052	0.0067	0.4413
			Weighted median	-0.0001	0.0017	0.9606
			Inverse variance weighted	0.0026	0.0020	0.1988
			Simple mode	0.0005	0.0033	0.8754
			Weighted mode	0.0005	0.0030	0.8619
Albumin	Exposure	130	MR Egger	-0.6775	0.6244	0.2801
			Weighted median	-0.7590	0.4418	0.0858
			Inverse variance weighted	-0.4864	0.2793	0.0815
			Simple mode	-1.4070	1.2379	0.2580
			Weighted mode	-1.8168	1.1469	0.1158
	Outcome	44	MR Egger	0.0018	0.0061	0.7750
			Weighted median	-0.0010	0.0018	0.5881
			Inverse variance weighted	-0.0006	0.0018	0.7508
			Simple mode	-0.0036	0.0041	0.3859
			Weighted mode	-0.0034	0.0037	0.3590
Alanine aminotransferase	Exposure	123	MR Egger	0.8396	0.6118	0.1725
			Weighted median	1.0285	0.4735	0.0299
			Inverse variance weighted	0.4225	0.2884	0.1429
			Simple mode	-0.1479	1.0285	0.8859
			Weighted mode	1.0844	0.5449	0.0488
	Outcome	44	MR Egger	-0.0092	0.0060	0.1348
			Weighted median	-0.0009	0.0015	0.5479
			Inverse variance weighted	-0.0026	0.0018	0.1449
			Simple mode	-0.0013	0.0029	0.6602
			Weighted mode	-0.0011	0.0025	0.6597
Aspartate aminotransferase	Exposure	137	MR Egger	1.1272	0.6385	0.0798
			Weighted median	0.9223	0.4883	0.0589
			Inverse variance weighted	0.4431	0.2838	0.1185
			Simple mode	-0.3137	1.1195	0.7797
			Weighted mode	1.0439	0.5703	0.0693
	Outcome	44	MR Egger	-0.0055	0.0062	0.3812
			Weighted median	-0.0015	0.0015	0.3429
			Inverse variance weighted	-0.0013	0.0018	0.4798
			Simple mode	-0.0020	0.0030	0.5173
			Weighted mode	-0.0025	0.0027	0.3558
Red blood cell (erythrocyte) count	Exposure	189	MR Egger	-0.0202	0.5010	0.9679
			Weighted median	-0.8140	0.3999	0.0418
			Inverse variance weighted	-0.4555	0.2355	0.0530
			Simple mode	-1.3524	0.8980	0.1337
			Weighted mode	-0.7856	0.5199	0.1324
	Outcome	43	MR Egger	0.0038	0.0045	0.3999
			Weighted median	0.0001	0.0015	0.9263
			Inverse variance weighted	0.0017	0.0013	0.1949
			Simple mode	-0.0014	0.0033	0.6743
			Weighted mode	-0.0009	0.0032	0.7763
Red blood cell (erythrocyte) distribution width	Exposure	152	MR Egger	-0.1667	0.4132	0.6871
			Weighted median	-0.5782	0.3445	0.0933
			Inverse variance weighted	-0.2920	0.2170	0.1784
			Simple mode	-1.1154	0.7883	0.1591
			Weighted mode	-0.9063	0.5415	0.0963
	Outcome	44	MR Egger	-0.0034	0.0072	0.6341
			Weighted median	0.0008	0.0017	0.6380
			Inverse variance weighted	0.0040	0.0021	0.0591
			Simple mode	0.0010	0.0033	0.7645
			Weighted mode	0.0006	0.0025	0.8135
Urea	Exposure	199	MR Egger	-0.2768	0.7610	0.7169

			Weighted median	0.2300	0.4945	0.6418
			Inverse variance weighted	-0.3032	0.3193	0.3422
			Simple mode	0.6339	1.1039	0.5671
			Weighted mode	0.0297	0.7766	0.9696
	Outcome	44	MR Egger	0.0022	0.0045	0.6328
			Weighted median	-0.0005	0.0016	0.7496
			Inverse variance weighted	0.0015	0.0013	0.2426
			Simple mode	-0.0015	0.0036	0.6758
			Weighted mode	-0.0011	0.0029	0.7172
Insulin-like growth factor 1	Exposure	183	MR Egger	0.1998	0.4770	0.6758
			Weighted median	-0.0648	0.3674	0.8599
			Inverse variance weighted	-0.1314	0.2169	0.5445
			Simple mode	0.1161	0.9975	0.9074
			Weighted mode	-0.2671	0.8390	0.7506
	Outcome	44	MR Egger	-0.0027	0.0064	0.6754
			Weighted median	-0.0015	0.0016	0.3525
			Inverse variance weighted	-0.0007	0.0019	0.7257
			Simple mode	-0.0002	0.0033	0.9395
			Weighted mode	-0.0013	0.0028	0.6381
Pain type(s) experienced in last month: Knee pain	Exposure	9	MR Egger	07.4722	16.8820	0.6714
			Weighted median	3.7904	5.1528	0.4620
			Inverse variance weighted	-0.1545	3.8506	0.9680
			Simple mode	8.2950	9.3229	0.3996
			Weighted mode	7.0612	8.3902	0.4245
	Outcome	43	MR Egger	0.0003	0.0016	0.8555
			Weighted median	0.0007	0.0006	0.2361
			Inverse variance weighted	0.0004	0.0005	0.4439
			Simple mode	0.0010	0.0012	0.4002
			Weighted mode	0.0010	0.0010	0.3624
Coffee intake	Exposure	18	MR Egger	-1.1622	1.7921	0.5259
			Weighted median	-0.3401	1.2181	0.7801
			Inverse variance weighted	0.1475	0.9527	0.8770
			Simple mode	0.1047	1.8716	0.9561
			Weighted mode	-0.2320	1.1271	0.8394
	Outcome	43	MR Egger	0.0002	0.0027	0.9329
			Weighted median	0.0015	0.0011	0.1810
			Inverse variance weighted	0.0013	0.0008	0.0996
			Simple mode	0.0026	0.0022	0.2361
			Weighted mode	0.0022	0.0020	0.2990
Sitting height	Exposure	344	MR Egger	-0.7572	0.5248	0.1500
			Weighted median	-0.2171	0.3765	0.5641
			Inverse variance weighted	0.1319	0.2268	0.5607
			Simple mode	0.5429	0.9835	0.5813
			Weighted mode	-0.3319	0.6340	0.6010
	Outcome	43	MR Egger	-0.0041	0.0047	0.3892
			Weighted median	-0.0013	0.0013	0.3285
			Inverse variance weighted	0.0010	0.0014	0.4502
			Simple mode	-0.0014	0.0032	0.6746
			Weighted mode	-0.0018	0.0039	0.6476
Age first had sexual intercourse	Exposure	120	MR Egger	0.4607	2.3558	0.8453
			Weighted median	-0.4365	0.6218	0.4826
			Inverse variance weighted	-0.9647	0.4266	0.0238
			Simple mode	-0.1264	1.7856	0.9437
			Weighted mode	-0.2481	1.8138	0.8914
	Outcome	43	MR Egger	-0.0015	0.0047	0.7462
			Weighted median	-0.0025	0.0015	0.0976
			Inverse variance weighted	-0.0033	0.0014	0.0149
			Simple mode	0.0019	0.0035	0.5859
			Weighted mode	0.0009	0.0030	0.7590
Average total household income before tax	Exposure	24	MR Egger	1.1996	3.7939	0.7548
			Weighted median	1.0259	1.0899	0.3466
			Inverse variance weighted	0.1801	0.7924	0.8202
			Simple mode	2.3475	1.9908	0.2504
			Weighted mode	1.9240	1.7200	0.2748
	Outcome	43	MR Egger	0.0050	0.0061	0.4220
			Weighted median	0.0001	0.0020	0.9624
			Inverse variance weighted	-0.0006	0.0018	0.7210
			Simple mode	0.0047	0.0039	0.2371
			Weighted mode	0.0011	0.0028	0.7013

Seated height	Exposure	328	MR Egger	-0.1347	0.4591	0.7694
			Weighted median	-0.1878	0.2972	0.5275
			Inverse variance weighted	-0.0786	0.1847	0.6704
			Simple mode	-0.4066	0.8011	0.6121
			Weighted mode	-0.3485	0.5177	0.5013
	Outcome	43	MR Egger	0.0050	0.0061	0.4220
			Weighted median	0.0001	0.0019	0.9621
			Inverse variance weighted	-0.0006	0.0018	0.7210
			Simple mode	0.0047	0.0042	0.2685
			Weighted mode	0.0011	0.0030	0.7245

3 Simulation Details and Results

3.1 Simulation settings

The simulated data were generated as follows. The n elements of the minor allele dose vector x were generated from a binomial distribution with two trials and the probabilities equal to a certain minor allele frequency (MAF). Then the trait vector y was generated from the multivariate normal distribution with mean vector $x\beta$ and covariance matrix $\mathbf{\Sigma}$. Binary traits were generated by determining whether y_i is greater than a cutoff in simulations. We let the β vector have p elements with d many non-zero elements. Larger β element values correspond to stronger associations between the traits and the SNP. The null SNPs for trait correlation estimation were simulated in the same way except the effect sizes are all zero. For the correlation structure, we tested homogeneous correlation (scenarios 1 and 3), real data variance-covariance matrix using the 754 UK Biobank traits (scenario 2), non-homogeneous correlation (scenario 4), and block-diagonal correlation scenarios (scenarios 5 and 6). Under the homogeneous correlation scenarios, all non-diagonal entries of the $\mathbf{\Sigma}$ matrix were equal to ρ, i.e., all pairs of traits had the same correlation. Diagonal entries of $\mathbf{\Sigma}$ are set to one. Under non-homogeneous correlation scenarios, half traits were negatively correlated with the rest, that is, for $i \neq j$, $\mathbf{\Sigma}_{ij} = -\rho$ if $i+j$ was odd, and $\mathbf{\Sigma}_{ij} = \rho$ otherwise. Within-block correlations were homogeneous, and inter-block correlations were zero. We simulated both individual-level data input and summary-level data input in scenario 1, and summary-level input only in the rest scenarios. For summary-level data input, we mimicked a typical GWAS, fitted linear regression for continuous traits and logistic regression for binary outcomes for each SNP x, and obtained their summary statistics.

In scenarios 1 to 5, we set sample size $n = 1,000$, and SNP of interest has a MAF$= 0.2$, number of null SNPs $= 2,500$, cutoff of binary outcome equals 0.2, and multiple correlation structures between traits to test their effects on performance. In scenario 6, we also kept the sample size $n = 1,000$ and MAF$= 0.2$. We conducted 1,000 runs of the simulations at each setting for power, and let $B = 10,000$ for the MC simulation step.

The detailed simulation settings are described below:

- Scenario 1: a total of 50 continuous traits with homogeneous effect sizes and correlation were simulated. The number of truly associated traits $||S_0||$ was varied at 1/2/4/6/8/22/36/50 where the effect size of S_0, β_t, was kept at 0.15, and correlation between any pair of traits was set to 0.2, i.e., $\sigma_{ij} = 0.2$.

- Scenario 2: variance-covariance matrix and effect sizes mimicking the UK Biobank data were simulated. S_0 contained 74 traits with raw p-values smaller than 0.05. The true effects were simulated by the estimated correlations between the traits and SNP rs6106336 from UK Biobank multiplied by a factor of 0/2/4/6/8/10 to evaluate method performance under different strengths of associations.

- Scenario 3: simulation setting is the same as scenario 1 except that half of the traits are binary. Meanwhile, half of the truly associated traits were continuous, and the other half were binary.

- Scenario 4: non-homogeneous effect sizes on 25 continuous traits and 25 binary traits with $||S_0|| = 4$ were simulated. Effect sizes of two truly associated traits (one continuous and one binary) $\beta_{t1} = 0.1$, while effect sizes of two others $\beta_{t2} = 0.2/0.3/0.4/-0.2/-0.3/-0.4$. $\sigma_{ij} = 0.2$.

- Scenario 5: 50 continuous traits with $\beta_t = 0.1$ and a block-diagonal correlation matrix were simulated. The 50 traits were grouped into four blocks which separately contain 5, 10, 15, and 20 traits. The within-block correlation $\sigma_{ij} = 0.2$ for i, j in the same block, and between-block correlation $\sigma_{ij} = 0$ for i, j in different blocks.

- Scenario 6: varying correlation magnitudes and directions on 25 continuous traits and 25 binary traits were simulated. Effect sizes of four truly associated traits (two continuous and two binary) $\beta_t = 0.15$. For settings with homogeneous correlations, $\sigma_{ij} = 0.2/0.5/0.8$ for each i, j. For settings with non-homogeneous correlations, $\sigma_{ij} = 0.2/0.5/0.8$ if $i + j$ is even, and $\sigma_{ij} = -0.2/-0.5/-0.8$ if $i + j$ is odd.

3.2 Supplementary simulation results

Table S3: TraitScan applied on individual-level and summary-level data in simulation scenario 1

$\|\|S_0\|\|$			1	2	4	6	8	22	36	50
Power	Summary level	TraitScan-HC	0.400	0.600	0.821	0.902	0.943	0.971	0.908	0.347
		TraitScan-TC	0.375	0.634	0.900	0.976	0.993	1.000	1.000	0.680
		TraitScan-minp:HC+TC	0.374	0.631	0.899	0.976	0.993	1.000	1.000	0.679
	Individual level	TraitScan-HC	0.445	0.651	0.836	0.926	0.954	0.983	0.938	0.384
		TraitScan-TC	0.435	0.679	0.915	0.979	0.996	1.000	1.000	0.725
		TraitScan-minp:HC+TC	0.434	0.678	0.914	0.979	0.996	1.000	1.000	0.724
Size	Summary level	TraitScan-HC	1.479	1.551	1.987	2.446	2.839	3.791	3.286	2.152
		TraitScan-TC	2.353	2.765	3.470	3.855	4.020	4.410	5.318	5.298
		TraitScan-minp:HC+TC	2.168	2.595	3.362	3.808	3.994	4.410	5.317	5.142
	Individual level	TraitScan-HC	1.463	1.583	2.039	2.548	3.000	4.108	3.501	2.175
		TraitScan-TC	2.475	2.773	3.351	3.681	3.874	4.098	4.444	5.581
		TraitScan-minp:HC+TC	2.290	2.627	3.257	3.632	3.853	4.097	4.442	5.450
Jaccard Similarity	Summary level	TraitScan-HC	0.609	0.529	0.428	0.370	0.327	0.146	0.059	0.043
		TraitScan-TC	0.490	0.535	0.536	0.477	0.407	0.163	0.095	0.106
		TraitScan-minp:HC+TC	0.524	0.532	0.528	0.473	0.405	0.163	0.095	0.103
	Individual level	TraitScan-HC	0.625	0.540	0.441	0.384	0.343	0.156	0.063	0.044
		TraitScan-TC	0.499	0.540	0.513	0.455	0.392	0.155	0.079	0.112
		TraitScan-minp:HC+TC	0.530	0.538	0.506	0.451	0.391	0.155	0.079	0.109
Precision	Summary level	TraitScan-HC	0.609	0.808	0.913	0.939	0.945	0.875	0.666	1.000
		TraitScan-TC	0.490	0.660	0.800	0.865	0.896	0.858	0.670	1.000
		TraitScan-minp:HC+TC	0.524	0.684	0.810	0.867	0.897	0.858	0.671	1.000
	Individual level	TraitScan-HC	0.625	0.814	0.914	0.938	0.942	0.863	0.661	1.000
		TraitScan-TC	0.499	0.674	0.813	0.876	0.904	0.862	0.662	1.000
		TraitScan-minp:HC+TC	0.530	0.696	0.822	0.879	0.904	0.862	0.662	1.000
Recall	Summary level	TraitScan-HC	0.734	0.577	0.443	0.378	0.333	0.149	0.061	0.043
		TraitScan-TC	0.807	0.738	0.632	0.525	0.433	0.169	0.100	0.106
		TraitScan-minp:HC+TC	0.789	0.713	0.618	0.520	0.431	0.169	0.100	0.103
	Individual level	TraitScan-HC	0.743	0.591	0.457	0.394	0.350	0.160	0.064	0.044
		TraitScan-TC	0.824	0.736	0.605	0.502	0.418	0.159	0.082	0.112
		TraitScan-minp:HC+TC	0.806	0.716	0.594	0.497	0.416	0.159	0.082	0.109

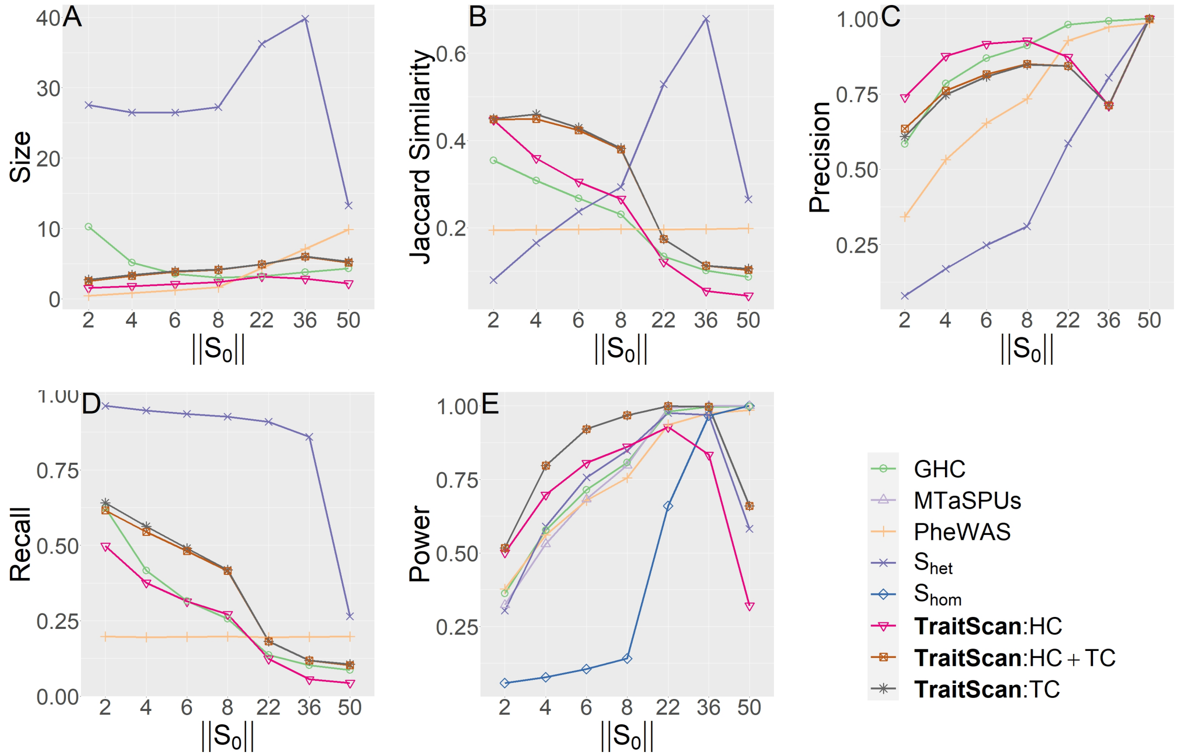

Figure S1: Simulations with continuous-binary mixed traits

Table S4: Simulation results for non-homogeneous effect sizes

Non-homogeneous Effect Sizes		0.2	0.3	0.4	-0.2	-0.3	-0.4
Power	PheWAS	0.741	0.996	1.000	0.755	0.994	1.000
	S_{hom}	0.078	0.095	0.118	0.057	0.063	0.071
	S_{het}	0.607	0.874	0.996	0.688	0.926	0.997
	MTaSPUs	0.697	0.993	1.000	0.715	0.995	1.000
	GHC	0.736	0.996	1.000	0.760	0.997	1.000
	TraitScan-HC	0.852	0.999	1.000	0.880	0.999	1.000
	TraitScan-TC	0.893	1.000	1.000	0.920	1.000	1.000
	TraitScan-minp:HC+TC	0.893	1.000	1.000	0.920	1.000	1.000
Size	PheWAS	1.072	1.944	2.130	1.040	1.900	2.122
	S_{het}	25.438	19.232	11.904	23.998	17.705	11.269
	GHC	3.219	1.133	1.046	2.770	1.128	1.036
	TraitScan-HC	1.691	2.129	2.213	1.721	2.176	2.306
	TraitScan-TC	2.877	2.161	2.155	2.860	2.194	2.235
	TraitScan-minp:HC+TC	2.801	2.159	2.155	2.797	2.194	2.235
Jaccard Similarity	PheWAS	0.255	0.471	0.517	0.247	0.46	0.515
	S_{het}	0.161	0.219	0.299	0.171	0.251	0.360
	GHC	0.288	0.278	0.262	0.298	0.277	0.259
	TraitScan-HC	0.363	0.497	0.515	0.384	0.515	0.547
	TraitScan-TC	0.428	0.497	0.512	0.455	0.515	0.539
	TraitScan-minp:HC+TC	0.424	0.497	0.512	0.451	0.515	0.539
Precision	PheWAS	0.716	0.98	0.986	0.732	0.979	0.986
	S_{het}	0.172	0.281	0.468	0.179	0.291	0.473
	GHC	0.876	0.997	1.000	0.901	0.997	1.000
	TraitScan-HC	0.920	0.966	0.965	0.939	0.973	0.974
	TraitScan-TC	0.797	0.961	0.976	0.825	0.97	0.983
	TraitScan-minp:HC+TC	0.805	0.961	0.976	0.831	0.970	0.983
Recall	PheWAS	0.257	0.475	0.522	0.249	0.464	0.520
	S_{het}	0.892	0.818	0.688	0.910	0.873	0.808
	GHC	0.344	0.279	0.262	0.346	0.278	0.259
	TraitScan-HC	0.375	0.507	0.526	0.394	0.523	0.556
	TraitScan-TC	0.501	0.509	0.520	0.523	0.524	0.545
	TraitScan-minp:HC+TC	0.493	0.508	0.520	0.516	0.524	0.545

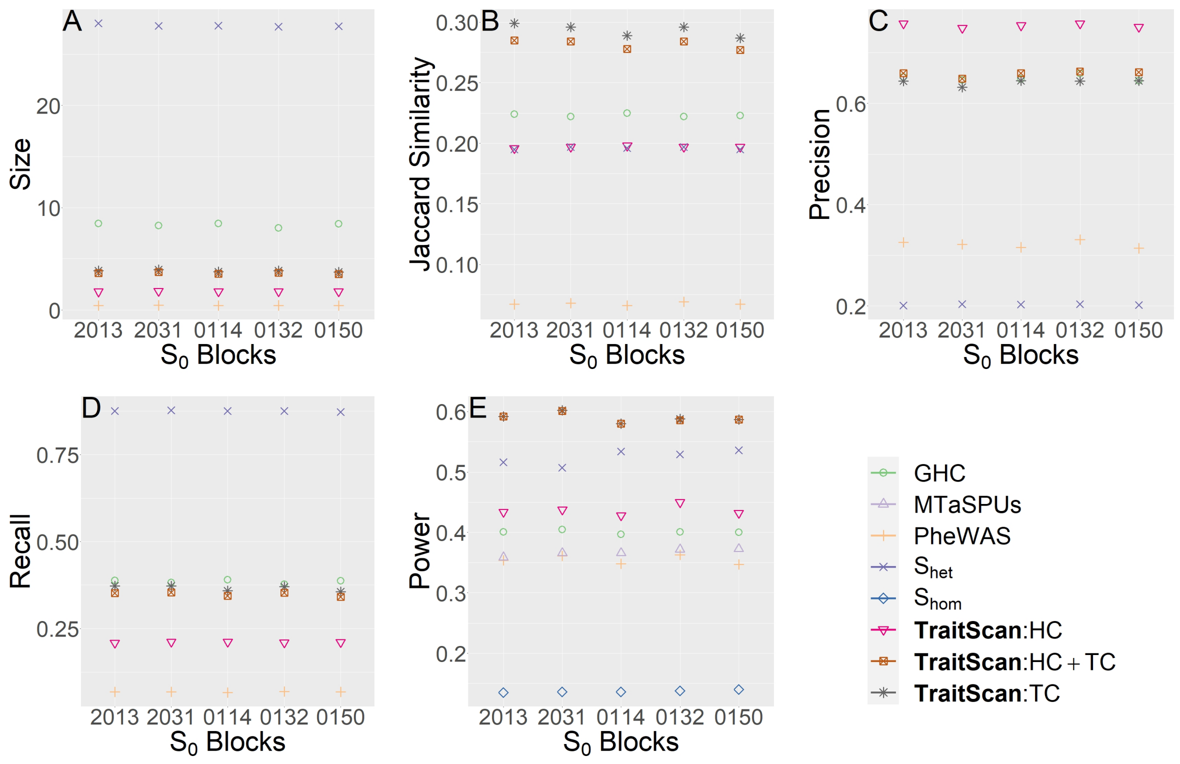

Figure S2: Simulations with block-correlated trait (x axis labels representing the numbers of truly associated traits in each block)

Table S5: Simulation results for different correlation magnitude and direction

Non-homogeneous Correlation		0.2	0.3	0.4	-0.2	-0.3	-0.4
Power	PheWAS	0.561	0.511	0.406	0.599	0.577	0.551
	S_{hom}	0.078	0.059	0.060	0.317	0.430	0.740
	S_{het}	0.590	0.656	0.681	0.651	0.730	0.869
	MTaSPUs	0.531	0.491	0.500	0.661	0.664	0.720
	TraitScan-HC	0.698	0.919	1.000	0.746	0.949	1.000
	GHC	0.577	0.429	0.266	0.613	0.479	0.326
	TraitScan-TC	0.797	0.960	1.000	0.831	0.974	1.000
	TraitScan-minp:HC+TC	0.797	0.960	1.000	0.831	0.974	1.000
Size	PheWAS	0.829	0.846	0.828	0.838	0.824	0.818
	S_{het}	26.461	27.056	25.694	25.009	23.886	18.521
	GHC	5.144	10.563	22.127	4.464	7.153	14.026
	TraitScan-HC	1.802	2.117	3.044	1.833	2.245	3.046
	TraitScan-TC	3.407	3.178	2.945	3.366	3.048	2.950
	TraitScan-minp:HC+TC	3.261	3.117	2.945	3.234	3.002	2.950
Jaccard Similarity	PheWAS	0.532	0.487	0.395	0.573	0.559	0.541
	S_{het}	0.169	0.208	0.280	0.175	0.230	0.399
	GHC	0.785	0.736	0.572	0.827	0.841	0.748
	TraitScan-HC	0.876	0.943	0.969	0.897	0.957	0.976
	TraitScan-TC	0.747	0.851	0.975	0.773	0.890	0.981
	TraitScan-minp:HC+TC	0.761	0.857	0.975	0.786	0.894	0.981
Precision	PheWAS	0.533	0.471	0.399	0.555	0.551	0.525
	S_{het}	0.166	0.207	0.275	0.173	0.228	0.397
	GHC	0.785	0.731	0.577	0.817	0.838	0.733
	TraitScan-HC	0.875	0.944	0.965	0.895	0.958	0.975
	TraitScan-TC	0.704	0.841	0.953	0.743	0.880	0.966
	TraitScan-minp:HC+TC	0.742	0.869	0.965	0.775	0.902	0.974
Recall	PheWAS	0.196	0.201	0.199	0.198	0.196	0.196
	S_{het}	0.947	0.939	0.904	0.95	0.949	0.932
	GHC	0.417	0.479	0.636	0.406	0.428	0.515
	TraitScan-HC	0.376	0.49	0.731	0.397	0.530	0.738
	TraitScan-TC	0.563	0.622	0.712	0.583	0.638	0.719
	TraitScan-minp:HC+TC	0.545	0.614	0.712	0.568	0.632	0.719

References

D. Bu, et al. Truncated tests for combining evidence of summary statistics. *Genetic Epidemiology*, 44(7):687–701, 2020.

D. B. Neill. Fast subset scan for spatial pattern detection: Fast Subset Scan. *Journal of the Royal Statistical Society: Series B (Statistical Methodology)*, 74(2):337–360, Mar. 2012. ISSN 13697412. doi: 10.1111/j.1467-9868.2011.01014.x. URL `https://onlinelibrary.wiley.com/doi/10.1111/j.1467-9868.2011.01014.x`.

X. Zhu, et al. Meta-analysis of correlated traits via summary statistics from gwass with an application in hypertension. *The American Journal of Human Genetics*, 96(1):21–36, 2015.
